# Immune checkpoint therapy responders display early clonal expansion of tumor infiltrating lymphocytes

**DOI:** 10.1080/2162402X.2024.2345859

**Published:** 2024-04-26

**Authors:** Joel Kidman, Rachael M. Zemek, John-William Sidhom, Debora Correa, Nicola Principe, Fezaan Sheikh, Vanessa S. Fear, Catherine A. Forbes, Abha Chopra, Louis Boon, Ayham Zaitouny, Emma de Jong, Robert A. Holt, Matt Jones, Michael J. Millward, Timo Lassmann, Alistair R.R. Forrest, Anna K. Nowak, Mark Watson, Richard A. Lake, W. Joost Lesterhuis, Jonathan Chee

**Affiliations:** a National Centre for Asbestos Related Diseases, Institute for Respiratory Health, University of Western Australia, Perth, Australia; b Telethon Kids Institute, Perth, Australia; c Icahn School of Medicine, Mt Sinai Hospital, New York, USA; d Complex Systems Group, Department of Mathematics and Statistics, University of Western Australia, Perth, Australia; e Medical Genomics Laboratories (IIID), Centre for Molecular Medicine and Innovative Therapeutics, Health Futures Institute, Murdoch University, Murdoch, Australia; f JJP Biologics, Warsaw, Poland; g Department of Mathematical Sciences, United Arab Emirates University, Al Ain, United Arab Emirates; h Medical School, University of Western Australia, Perth, Australia; i BC Cancer Research Institute, Vancouver, Canada; j Harry Perkins Institute of Medical Research, QEII Medical Centre and Centre for Medical Research, The University of Western Australia, Perth, Australia

**Keywords:** Immune checkpoint therapy, T cells, T cell receptor (TCR) sequencing, tumour-infiltrating lymphocytes, immune repertoire, machine learning

## Abstract

Immune checkpoint therapy (ICT) causes durable tumour responses in a subgroup of patients, but it is not well known how T cell receptor beta (TCRβ) repertoire dynamics contribute to the therapeutic response. Using murine models that exclude variation in host genetics, environmental factors and tumour mutation burden, limiting variation between animals to naturally diverse TCRβ repertoires, we applied TCRseq, single cell RNAseq and flow cytometry to study TCRβ repertoire dynamics in ICT responders and non-responders. Increased oligoclonal expansion of TCRβ clonotypes was observed in responding tumours. Machine learning identified TCRβ CDR3 signatures unique to each tumour model, and signatures associated with ICT response at various timepoints before or during ICT. Clonally expanded CD8+ T cells in responding tumours post ICT displayed effector T cell gene signatures and phenotype. An early burst of clonal expansion during ICT is associated with response, and we report unique dynamics in TCRβ signatures associated with ICT response.

## Introduction

Drugs that target immune checkpoint receptors on T cells, such as programmed death (PD)-1 and cytotoxic T lymphocyte-associated antigen (CTLA)-4, are approved for the treatment of multiple cancers, with durable responses observed in a subset of patients.^1,2^ However, not all patients treated with immune checkpoint therapy (ICT) benefit. Tumours with high PD-L1 expression, high mutational burden and increased lymphocytic infiltration are more likely to respond to ICT.^3–5^ However, there is no single accurate biomarker of ICT response that can be applied across cancers.^6^ As ICT primarily acts upon adaptive immunity, in-depth profiling of T cell phenotype and specificity in responders and non-responders to therapy improves development of predictive biomarkers for response.^7–9^

High throughput sequencing of TCRα/β chains (TCRseq) has been widely used to profile the distribution of TCR clonotypes within tumor and peripheral blood samples in relation to ICT outcomes.^10^ For example, anti-PD-1 therapy drives clonal expansion of antigen-specific T cells, reflected by more clonal TCRβ repertoires of tumor-infiltrating lymphocytes (TILs).^11–13^ A pre-PD-1 treatment, clonal TIL TCRβ repertoire differentiated response in some studies, but not others.^11,14^ In terms of peripheral blood, a more diverse pre-anti-PD-1 treatment TCRβ repertoire was associated with response but a more clonal TCRβ repertoire predicted increased overall survival for patients receiving anti-CTLA4 therapy.^15^ Peripheral blood expansion of tumor-antigen-specific T cell clones over the course of PD-1 treatment occurred in responding patients.^16^  Other advances in the field include sequence-based clustering of TCRs to identify common motifs predictive of specificity to the same antigen.^17,18^ Despite these advances, the value of TCR repertoire profiling as a predictive biomarker is limited in the context of ICT. The inability to identify clear TCR correlates with response may be because clinical studies are confounded by variability in host genetics, tumor heterogeneity and environmental factors. Furthermore, frequent serial tumor biopsies are not feasible in clinical studies, making it difficult to assess TCRβ repertoire dynamics within the tumour microenvironment.

To study TCRβ repertoire dynamics in relation to ICT response, we leveraged murine models with limited genetic, antigenic and environmental variation, allowing us to examine tumors and blood before and after ICT.^19,20^ By applying bulk TCRβ sequencing, single cells sequencing and machine learning, we interrogated TCRβ in pre- and post-ICT samples to determine if there were features of the TCRβ repertoire associated with therapy response. Changes in overall tumor TCRβ diversity, and TCRβs signatures unique to each tumor model delineated ICT responders from non-responders.

## Methods

### Mice

BALB/c and Clone 4 (CL4xThy1.1) mice were bred and maintained at the Animal Resource Centre (Murdoch, Australia) or Harry Perkins Medical Research Institute (Nedlands, Australia). CL4×Thy1.1 TCR transgenic mice express a TCR that recognizes a MHC class I-restricted influenza A/PR/8 hemagglutinin (HA_533–541_) epitope. CL4 T cells expressed allelic marker Thy1.1. Female mice aged between 8 and 10 weeks of age were used for experiments. All animal experiments were carried out in accordance with approved Harry Perkins Institute of Medical Research Animal Ethics guidelines.

### Adoptive transfer of TCR transgenic splenocytes

Spleens from CL4×Thy1.1 mice were manually dissociated through 40 µm strainers with phosphate-buffered saline (PBS) supplemented with 2% Newborn Calf Serum (NCS; Life Technologies). Red blood cells were lysed with Pharm Lyse (BD Biosciences) and splenocytes were washed twice with PBS. BALB/c recipients were intravenously injected with 1 × 10^5^ cells where indicated.

### Tumour cell lines and inoculation

Murine malignant mesothelioma cell line AB1 and AB1 cell lines transfected with the influenza hemagglutinin (HA) from PR8/24/H1N1 strain (AB1-HA) were generated and maintained as previously described.^21^ Murine renal cell carcinoma RENCA was obtained from ATCC. For all bilateral tumor experiments, the shaved right and left flanks of mice were inoculated subcutaneously with 5 × 10^5^ tumor cells in each flank.^20^ The same number of cells was inoculated only into the shaved right-hand flanks for single flank experiments.

### Immune checkpoint blockade (ICT) therapy

All ICT antibodies were administered by intraperitoneal injection. Combination anti-CTLA-4 clone 9H10 (100 μg) and anti-PD-L1 clone MIH5 (100 μg) was administered when tumors were 10–15 mm^2^, with two additional doses of anti-PD-L1 (100 μg) every two days. All treatments were randomized and blinded. Mice were defined as responders when their tumor completely regressed and remained tumor-free for at least 4 weeks after treatment. Mice were designated as non-responders if their tumors grew to >100 mm^2^ within 4 weeks after the start of therapy. Where indicated, three doses of combination anti-CTLA-4 (100 μg) and anti-OX-40 clone OX-86 (200 μg) were administered three days apart via intraperitoneal injection, starting when tumors were 20–30 mm^2.22^

### Tumour debulking surgery and blood collection

Complete tumor debulking of the right flank was performed on the day prior to anti-CTLA-4 and anti-PD-L1(pre-treatment), 2 days (+2), 4 days (+4) or 6 days (+6) post the first dose of treatment as previously described.^20^ Briefly, whole tumors were surgically excised, and wounds closed when mice were under anesthesia. In a different series of experiments, peripheral blood samples from animals were collected one day prior to anti-CTLA-4 and anti-PD-L1(pre-treatment), 3 days (+3) and 6 days (+6) post first dose of treatment.

### Tissue processing

Tumours were dissociated using gentleMACS (Miltenyi Biotec) as per manufacturer’s instructions. Dissociated tumor tissue was stored in Qiagen RNAprotect Cell Reagent (Qiagen #76526) prior to RNA extraction. Heparinized peripheral blood was incubated with red blood cell lysis buffer for 5 minutes at room temperature. Samples were washed twice in PBS + 2% NCS and stored in Qiagen RNA Cell Protect prior to RNA extraction.

### Fluorescence activated cell sorting

Single cell suspensions were stained for 30 minutes with CD4[BV421] (clone GK1.5), CD45[APC] (clone 30-F11), CD8[APC-eFlour780] (clone 53–6.7), CD3[PE-Cy7] (clone 17A2) antibodies, and a viability dye[efluor450] (Invitrogen). Live CD45^+^CD3^+^CD4^+^ or Live CD45^+^CD3^+^CD8^+^ cells were sorted on a BD FACS Melody cell sorter.

### Flow cytometry analysis

Flow cytometry panels outlined in supp table S1 were used to characterize T cell subsets. Zombie UV™ (BioLegend) viability dye was diluted in PBS and added to samples prior to surface antigen staining. All antibodies for surface staining were diluted in PBS + 2% NCS. Cells were permeabilized using the Foxp3/Transcription Factor Staining Buffer Set (eBioscience). Cells were washed with Permeabilization Buffer (eBioscience) and subjected to intracellular staining. Single stain and fluorescence minus-one (FMO) controls were also performed. Data was acquired using a BD LSRFortessa™ SORP with 20,000 T cell events collected per sample where possible. All flow cytometry analyses were completed using FlowJo™ Software version 10 (BD Biosciences), gating strategy displayed (Supp Figure 1).

**Figure 1.**
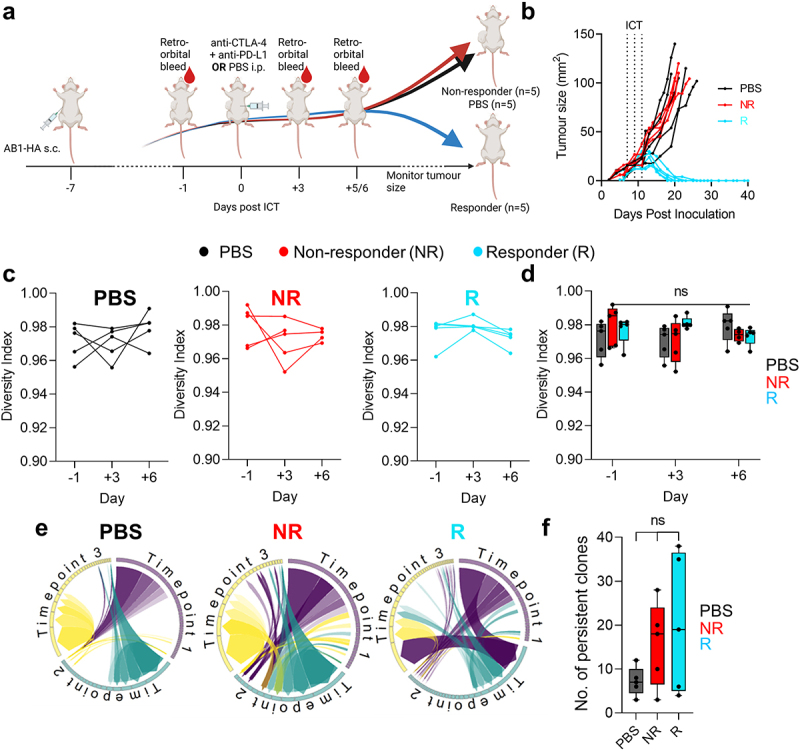
Dynamics in blood TCRβ repertoires were similar between ICT responders and non-responders. (a) Experimental timeline depicting when tumors were inoculated subcutaneously (s.c.), ICT (anti-CTLA-4 + anti-PD-L1) was treated via intraperitoneal (i.p.) injection and blood samples were acquired. (b) AB1-HA tumor growth curves of PBS and ICT treated animals. PBS (black, *n* = 5) treated, ICT responding (blue, *n* = 5) and non-responding (red, *n* = 5) animals represented. Dotted lines on growth curves represent when ICT was administered. (c, d) Change in TCRβ repertoire diversity in sequential blood samples from individual animals, represented by Shannon’s diversity indices. (e) Circos plots represent the distribution of most abundant clones across 3 timepoints. Each colored trine represents one time point, and connecting bands represent individual TCRβ clones. The width of each band connecting the trines depicts the frequency of that clone at both time points. (f) Number of persistent and abundant TCRβ clones, defined as any TCRβ clones that ranked top 100 in abundance at more than 1 timepoint. All analysis was performed on down-sampled data to ensure that repertoire sizes were similar. One-way ANOVA with Kruskal-Wallis tests were used to compare between groups at each time-point. One-way ANOVA with Friedman tests were used to compare TCRβ repertoires in individual animals over time. *p < 0.05.

### Bulk TCRβ sequencing

RNA extraction was performed using Qiagen RNeasy Plus Micro kits. TCRβ amplicon libraries were generated as previously described.^23^ 1 μg of sorted T cell or bulk tumor RNA was reverse transcribed to cDNA using SMARTerIIA-PID 5’RACE and TCRβ constant region 3’ primers. Primer IDentifiers (PIDs) account for sequencing error and amplification bias in downstream PCRs by creating a unique identifier for each RNA transcript that can be deconvoluted during TCRseq pre-processing. The 5’ Illumina sequencing adaptor was incorporated in a PCR using Roche KAPA HiFi HotStart ReadyMix (KK2602). 3’ Illumina sequencing adaptor was incorporated in a second PCR reaction. cDNA was purified between reactions using Beckman Coulter AMPure XP magnetic beads. 300bp paired end sequencing was performed on an Illumina HiSeq at the Medical Genomics Laboratories (Murdoch, WA, Australia).

### TCRβ sequencing data analysis

Aggregation of PIDs and alignment of CDR3 sequences to the IMGT/V-QUEST reference genome were performed using MiXCR pipelines.^24^ Only sequences with UMIs were aligned. TCRβ CDR3s containing fewer than 8 or more than 20 amino acid residues were excluded from analysis. Clonal T cells are defined by distinct TCRβ CDR3 amino acids. TCRβ CDR3 sequence for the CL4 clone (CASGETGTNERLFF) was previously determined.^23^ Repertoires were down sampled using rarefaction to the smallest sample to ensure comparability using the R package vegan as previously described.^25^ All diversity and dissimilarity indexes were computed on down sampled data using the R package vegan. Normalized Shannon’s diversity was used to measure distribution of clones, where higher values indicate a more diverse repertoire and lower values indicate a more clonally expanded repertoire. Renyi diversity is a parametrization of Shannon’s diversity that uniquely defines the distribution of clones in a repertoire graphically. Morisita-Horn index was used for pairwise comparison of TCRseq repertoires, where a maximum value of 1 indicates both repertoires are identical and 0 where they have no clones in common.

### Neural network based TCRβ clustering

We applied DeepTCR (v2.1.6), a deep learning framework for revealing sequence concepts within T cell repertoires.^26,27^ To identify a predictive signature of ICT response in the TCR repertoire of the tumor microenvironment, we fit the repertoire classifier on bulk TCRseq data using TCR sequence information (CDR3-β and V/D/J genes) from different timepoints. The repertoire classifier utilized a cross-validation strategy to establish the strength of the predictive signature for both tumor model and response-related model. DeepTCR was used to train a signature predictive of tumor model and ICT response from a subset of the total data, or data from a single timepoint. The predictive capacity of trained signatures was tested by applying the signature on data from the remaining samples. To assess the stability of the learning model, permutation tests were conducted by shuffling the sample labels. Details of machine learning performed in this study are reported as per DOME guidelines (Supp Table S2).^28^

### Single cell sequencing

50,000 to 100,000 CD45^+^ cells from each tumors were sorted. Sorted cells underwent methanol fixation, as previously described,^29^ prior to library preparation. All single-cell libraries were constructed using the 10× Chromium 5′ workflow as per the manufacturers’ directions. All libraries were quantified with qPCR and checked for fragment size. The libraries were pooled in equimolar concentration for a total pooled concentration of 2 nM. 10× single-cell libraries were sequenced using the Illumina NovaSeq 6000 and S2 flow cells.

### scRNA-seq data processing

Cell ranger (10× Genomics) was used to process the data from the 10X–5’ single cell RNA-seq and TCR-seq experiments using mouse reference mm10. Gene counts were normalized with Seurat v3.1 using SCtransform and scale-factor transform methods. Low quality cells that had either greater than 10% mitochondria content or less than 500 UMIs were filtered. Seurat’s CellCycle Scoring function was used for determining cell-cycle phases. Identification of clusters of single cells was performed by dimensional reduction using PCA and by applying graph clustering algorithms to the reduced components. Visualization of the results was performed with uniform manifold approximation and projection (UMAP) and t-distributed stochastic neighbor embedding (t-SNE). The clusters were annotated by cell types derived using ScMatch and SingleR with the help of FANTOM5 reference datasets. Joint analysis of merged samples was performed using R package Harmony. Differentially expressed genes (DEGs) between responders and non-responders for T cell subsets were derived based on log normalized average gene expression, with Benjamini-Hochberg adjusted *p* value less than 0.05.^30^ Pre-ranked gene set enrichment analysis (GSEA) was used to compare enrichment of gene signatures between responders and non-responders.^31^ CD8^+^ T cell exhaustion or memory signatures were derived from previous published studies.^32–34^ Gene sets enriched with an FDR score < 0.25 were considered significant.

### Data availability

Sequencing data are available under GEO accession number GSE222575. All hyperparameters for machine learning can be found in https://github.com/22461922Joel/Murine-tumour-dynamics-DeepTCR-2022.

### Statistics

All statistical tests were performed in R or in GraphPad Prism, details of each test in figure legends. Figures were made with BioRender.com.

## Results

### Early changes in blood TCRβ repertoire diversity do not correlate with ICT outcome

It is not clear whether there is a relationship between the available peripheral blood T cell repertoire and an ability to respond to ICT. Clinical TCRβ sequencing studies are limited by heterogeneity in tumor antigen expression, patient HLA diversity, differences in anti-viral TCRβ repertoire, all of which could affect the interpretation of TCRβ diversity and clonality. We used a murine model to exclude variation in tumor antigen load (tumors were derived from clonal cancer cell line AB1-HA), host genetics and MHC haplotype (inbred BALB/c strain of one gender), environmental factors (mice were kept under highly controlled conditions) and treatment schedule (all mice were treated identically). As reported before, despite this homogenous background, mice still separated into responders and non-responders.^19,20^ An important reason for this dichotomy in response could be the variability in TCR repertoire between individual mice. In this experiment, we therefore interrogated blood TCRβ repertoire following treatment with antibodies targeting CTLA-4 and PD-L1. We performed bulk TCRβ sequencing on sequential blood samples from a cohort of tumor bearing animals 1 day prior to, 3 and 6 days after the start of ICT (Figure 1(a)). We chose these early time points because key changes in peripheral blood T cells occur early after the commencement of immunotherapy.^35–37^ Importantly, tumor sizes were identical at these time points, regardless of eventual response (Figure 1(b)). We found that the distribution of TCRβ clones in peripheral blood, as represented by Shannon’s diversity index, was high (0.97 ± 0.1), suggesting that TCRβ clones were mostly evenly distributed within mice, with no clone dominating any repertoire (Figure 1(c)). The number of total and unique TCRβ sequences were likewise similar between responders and non-responders at all time points (Supp Figure S2(a,b)). There were also no patterns of change in TCRβ repertoire diversity associated with response, as individual animals maintained high diversity after ICT (Figure 1(c,d)). To determine if ICT resulted in an increase of TCRβ clones that persisted in peripheral blood, we enumerated abundant TCRβ clones that were present at all timepoints for each animal (Figure 1(e)). There were no differences in the number of persistent clones between responders and non-responders (Figure 1(f)). We tracked endogenous TCRβs (Clone 4, CL4) specific for the model antigen hemagglutinin (HA), because HA was expressed on the tumor cells.^21,38^ Our previous research demonstrated that an increase in CL4 T cell clones within the tumor, as measured by flow cytometry or the frequencies of CL4 TCRβ sequences, predicted ICT response in AB1-HA model.^23^ CL4 TCRβs were present at low frequencies in the blood (0.0% − 0.4%), and changes were not associated with ICT response (Supp Figure S2C). Together, these data show that overall TCRβ diversity in the peripheral blood did not differentiate responders from non-responders in our models.

### Bilateral tumours from the same animal exhibit highly similar TCRβ repertoires

**Figure 2.**
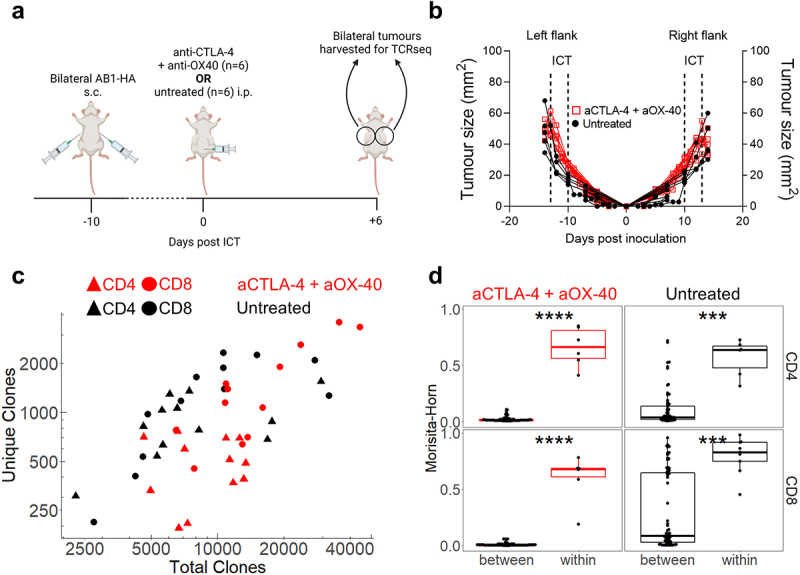
Bilateral tumors have highly similar TCRβ repertoires. (a) Experimental timeline, and (b) Tumor growth of PBS (black) and ICT (anti-CTLA-4 + anti-OX-40, red) treated AB1-HA bearing animals. Dotted lines represent treatment. Animals were euthanized so that tumors were size matched (*n* = 6/group). (c) Number of total and unique TCRβ clones derived from bulk TCRβ sequencing of sorted CD4^+^ (filled triangles) and CD8^+^ (filled circles) TILs from ICT (red) and PBS (black) treated animals (d) Morisita-Horn Overlap index values comparing down-sampled TCRβ repertoires of bilateral tumors within animals (*n* = 6 pairwise comparisons), or tumors between different animals (*n* = 60 comparisons). CD4^+^ and CD8^+^ TILs, ICT treatment plotted. All dot plots represent mean ± SEM. Mann-Whitney test used to compare different treatment groups. *p < 0.05, ***p < 0.005.

Since the overall TCRβ diversity in peripheral blood did not correlate with response to ICT, we queried whether that was due to the compartment examined. Therefore, we examined changes in TCRβ repertoires of TILs. We previously established a bilateral tumor model, whereby tumors on contralateral flanks of an animal respond symmetrically following the administration of ICT, allowing us to surgically excise one tumor to examine the TCRβ repertoire and leave the remaining tumor as a readout for response.^20^ We first examined whether TCRβ repertoires in symmetrical, bilateral tumors without surgery have similar distributions of identical TCRβ clones. We performed bulk TCRβ sequencing on sorted CD4^+^ and CD8^+^ TILs from bilateral AB1-HA tumors treated with anti-CTLA-4 and anti-OX-40 ICT because we previously observed robust anti-tumor effects with this combination (Figure 2(a,b)).^22^ Bilateral tumors were harvested when sizes between ICT and control groups were similar for both flanks (Figure 2(b), Supp Figure S3A). The number of total and unique TCRβ sequences from CD4^+^ and CD8^+^ TILs were similar between untreated and ICT treated animals, and between both flanks of the same animal (Figure 2(c), Supp Figure S3B, S3C). Next, we compared the TCRβ repertoires of bilateral tumors within the same animal, and between different animals, using the Morisita-Horn overlap index, which accounts for the number of identical TCRβ clonotypes and their distribution across each paired comparison. Regardless of treatment, CD4^+^ and CD8^+^ TCRβ repertoires in tumors were highly related within animals (0.64 ± 0.20), but not between animals (0.09 ± 0.19) (Figure 2(d)). To confirm that this similarity is maintained with a different combination ICT, (anti-CTLA-4 and anti-PD-L1), we performed bulk TCRβ sequencing on tumours from treated animals (Supp Figure S3(d)) and similarly found that TCRβ repertoires in tumours were highly related within animals, but not between animals (Supp Figure S3E). Importantly, these data demonstrate that within an individual mouse, the T cell repertoire of one flank tumor is highly representative of the other flank tumor giving us a reliable and valid readout of intra-tumoral TCRβ repertoire changes in relation to ICT outcomes for the following experiments that involved surgical excision of tumors.

### ICT responding tumours exhibit early clonal expansion of unique TCRβ clones

**Figure 3.**
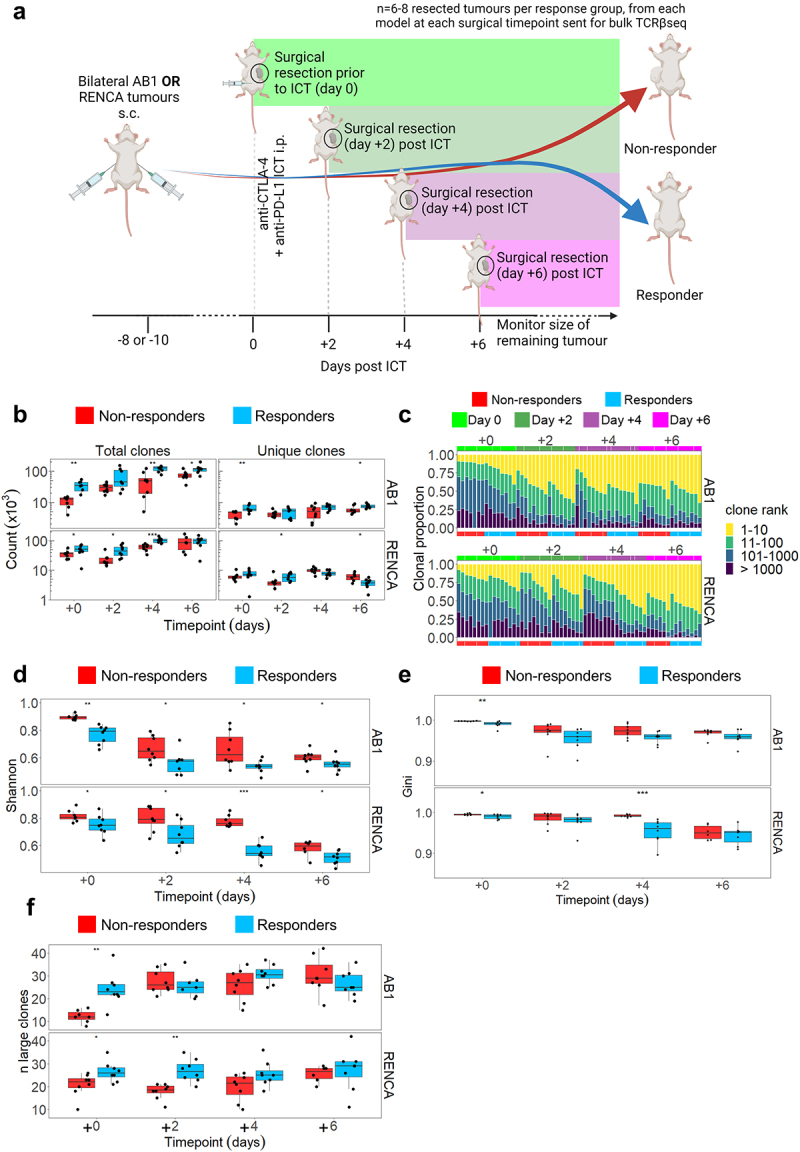
ICT responders display clonal expansion of unique tumor TCRβs earlier than non-responding mice. (a) Mice with bilateral AB1 or RENCA murine tumors were administered anti-CTLA-4 and anti-PD-L1 ICT. Each group had right flank tumors surgically excised before ICT, or every two days after ICT. 8 mice for each response group at each time point in each model, with the exception of day + 6 group in RENCA model (*n* = 6). (b) Number of total and unique TCRβ clones in tumors from responding (blue) and non-responding (red) mice across 4 timepoints. (c) TCRβ clones were ranked on their abundance within each tumor repertoire, and proportions of ranked TCRβs were plotted in relation to time and response. (d) Tumour TCRβ diversity represented by a normalized Shannon’s index in responding or non-responding mice. Higher indices indicate a more diverse repertoire and lower values correspond to more clonal repertoires. (e) Tumour TCRβ diversity represented by a Gini index in responding or non-responding mice. Gini index is more sensitive to high abundance clones, values close to 1 represent higher diversity and values closer to 0 represent lower diversity. (f) The number of large TCRβ clones (occupying >0.5% of each repertoire) compared between ICT response, and tumor models across time. All graphs from C to F display analyses performed on down-sampled data. All box plots depict mean ± SEM of individual animals, *n* = 6–8 mice/group. Mann-whitney test used to compare diversity index between responders vs non-responders, with Holm-Bonferroni method used to correct for multiple comparisons. *p < 0.05, ***p < 0.005, ****p < 0.001.

We characterized tumor TCRβ repertoires over time in relation to ICT outcomes in the next series of experiments. In the bilateral tumor model, we excised one flank tumor before (day 0), or 2, 4 or 6 days after anti-CTLA-4 and anti-PD-L1 treatment for bulk TCRβ sequencing in two mouse models, AB1 mesothelioma and RENCA renal cancer (Figure 3(a)). We consider these as early timepoints because tumor sizes are indistinguishable between response groups; typically, tumors in responders start to regress from day 7 post-ICT onwards in these models.^20,39^ We previously show that tumor sizes at the start of treatment were not different between responders and non-responders, nor between resected and remaining tumors.^20,39^ We previously characterized the transcriptome of these tumors, and in this study we performed bulk TCRβ sequencing on the samples. All tumors yielded between 10^3^ − 10^5^ total TCRβ clones and between 10^2^ − 10^4^ unique TCRβ clones (Figure 3(b), Supp Figure S4A). The total number of TCRβ clones increased over time in both models, suggesting that ICT increased the number of TILs. Responding tumors had a greater number of total TCRβ clones compared to non-responding tumors (all timepoints *p* < 0.05 except day 2 in AB1, and day 6 in RENCA), while the number of unique TCRβ clones remained similar between responders and non-responders at all time-points with the exception day 0 and 6 in AB1, and day 2 and 6 in RENCA (Figure 3(b)).

We next examined how ICT changed the diversity and clonality of T cells within each tumor. We measured TCRβ diversity and clonality with Rényi entropy and normalized Shannon’s index, which summarizes the proportional effect of each clone on the repertoire (Supp Figure S4B). Most of the pre-treatment tumor T cells were not highly expanded clones, with the top 10 most abundant clones occupying 18.9% ± 8.2% of repertoires (Figure 3(c)). Diversity, as represented by the normalized Shannon’s and Gini indices, decreased over time following ICT, with the top 10 most abundant clones occupying 49.6% ± 9.0% of the repertoires and the lowest diversity indices measured 6 days after treatment (Figure 3(c–e), Supp Figure S4C). Importantly, responding tumors had significantly lower TCRβ diversity at all timepoints as measured by normalized Shannon’s Index (Figure 3(d)). In both models, tumors from responding animals displayed TCRβ clonal expansion earlier in time as the number of highly expanded clones was significantly higher in responders prior to treatment (day 0) (Figure 3(f)). The number of highly expanded clones (that occupied more than 0.5% of the whole repertoire) remained stable after ICT (Figure 3(f)). By making pairwise comparisons across all animals, we found low Morisita-Horn’s overlap indices (ranging from 0.0003–0.16), indicating that tumor TCRβ repertoires from individual animals consisted of clones that were rarely shared with other animals (Supp Figure S4D). Taken together, our data suggests that ICT responding tumors have more T cell clones that expanded more rapidly after treatment compared to non-responders.

### Neural network identifies tumour-related and ICT response related TCRβ signatures at selected timepoints

**Figure 4.**
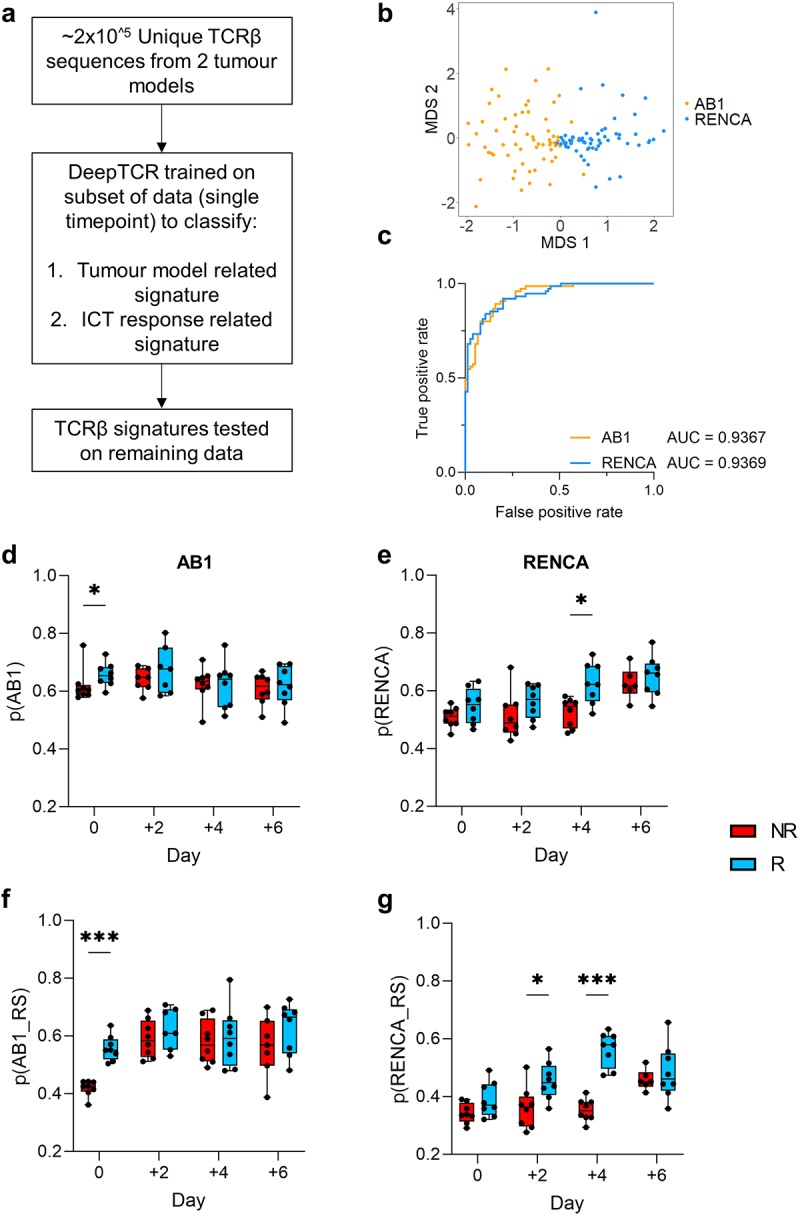
ICT response-related signatures can be trained from selected timepoints. (a) Schematic of DeepTCR classification of tumor TCRβs into ICT response and tumor model-related signatures (b) Unsupervised classification all unique TCRβ sequences in both models reduced data to 87 clusters. Multi-dimensional scaling plot of overall cluster expression, each dot represents one animal. AB1 (orange) or RENCA (blue) tumors overlayed in color; (c) ROC curve depicting how DeepTCR classified tumor-related signatures trained from day 0 samples are predictive of AB1 and RENCA tumor models when applied to remaining samples. (d, e) Modelling of tumor-related signatures, or (f, g) ICT-response-related signatures in samples over time, responders (blue, R) versus non-responders (red, R). Y-axis depicts weighted proportion of tumor-related signature in each sample. Mean±SEM depicted for each bar graph. *n* = 6–8 mice per group. Kruskal Wallis tests with multiple comparisons used to compare between timepoints. Multiple Mann-Whitney tests used to compare between responder and non-responder groups at individual timepoints. *p < 0.05, ***p < 0.0005.

We next investigated whether there were TCRβ sequence signatures from the bulk TCRβ sequencing data that were enriched in ICT responders. We applied a deep learning method (DeepTCR) that classifies TCRβs based on patterns such as CDR3 amino acid motifs and V/D/J gene usage (Figure 4(a)). It previously identified TCRβ signatures shared by T cells specific for the same antigen, and importantly derived TCRβ signatures predictive of ICT response in patient cohorts.^26,27^

We assumed that TCRβs from different mice harboring the same tumor would be composed of similar residue sequences because of their specificity against shared tumor-antigens.^40^ Unsupervised DeepTCR classified ~ 2×10^5^ unique TCRβs from both models into 87 final clusters, with each cluster having a median of 5710 unique TCRβ sequences. Samples primarily grouped by tumor models (AB1 vs RENCA) based on the expression of these TCRβ clusters (Figure 4(b)), suggesting that TCRβs from each tumor model were distinct. To perform more stringent cross validation, we next applied a supervised repertoire classifier to train a TCRβ signature predictive of tumor models with samples from day 0 (pre-ICT) only. We trained the model on this subset, and tested the derived TCRβ signature on the remaining data from other timepoints. We found TCRβs that classified the respective tumor models, which we term as tumor-related TCRβs (Supp Figure S5A, Supp Table S3). Tumor-related TCRβ signatures derived from the day 0 training set also predicted AB1 and RENCA TCRβ repertoires in the remaining samples from the other timepoints (Figure 4(c), Supp Figure S5B). To account for possible confounding effects, we applied the supervised repertoire classifier to samples with permutated labels. By randomly shuffling labels associated with tumor model and response outcomes for all samples whilst preserving the TCRβs within each sample, we conducted the supervised repertoire classification for each timepoint again. We did not observe a signature for tumor model identification in this permutation analysis, indicating the robustness of the classifier and confirming its ability to distinguish genuine tumor-related patterns from random label assignments (Supp Figure S5C).

To understand how tumor-related TCRβ signatures change over time in relation to ICT response, we assigned a probability to every TCRβ in our dataset for being AB1 or RENCA related, and quantified the tumor-related signature at each time point.^26^ In the AB1 model, the tumor-related signature was significantly higher in responders compared to non-responders prior to treatment (*p* = 0.04) (Figure 4(d)). The tumor-related signature remained stable in both responders and non-responders after treatment. In the RENCA model, the tumor-related signature increased over time (*p* = 0.0008), with the largest difference between responders and non-responders found on day 4 after treatment (*p* = 0.01) (Figure 4(e)).

We next applied the repertoire classifier to train a TCRβ signature to classify response and non-response in each model. We trained an ICT response signature using data from samples within each individual timepoint. In the AB1 model, a TCRβ signature that could classify ICT response could be trained from day 0 data (Supp Figure S5D). In the RENCA model, the strongest signature of ICT response was trained from day 4 post-ICT data (Supp Figure S5E). We validated the trained signature on data from other timepoints and found that the ICT response signature trained was weakly predictive at other timepoints for AB1 (Supp Figure S5F), and response signature trained RENCA day 4 data was predictive of response most other timepoints (Supp Figure S5G).

By quantifying the ICT response signature over time, we found that the ICT response signature was strongest in responders compared to non-responders at day 0 for AB1 (*p* = 0.0001) (Figure 4(f)), but not at any other timepoints. Conversely, the ICT response signature for RENCA was strongest after ICT, with significant differences in the signature found on day 2 and 4 (*p* = 0.02, *p* = 0.0001 respectively) (Figure 4(g)). To address whether tumor-related and ICT response signatures overlap, we found that the probabilities of individual TCRβs being tumor-related were also significantly correlated with the probabilities of being response related in both models (Supp Figure S5H, I). TCRβ signatures associated with ICT response could be only trained at some timepoints prior to, or post ICT. Differences in both models highlight the complexity and need to understand TCRβ dynamics in relation to ICT response.

### Expanded CD8^+^ T cell clones from responding tumours displayed an effector phenotype

**Figure 5.**
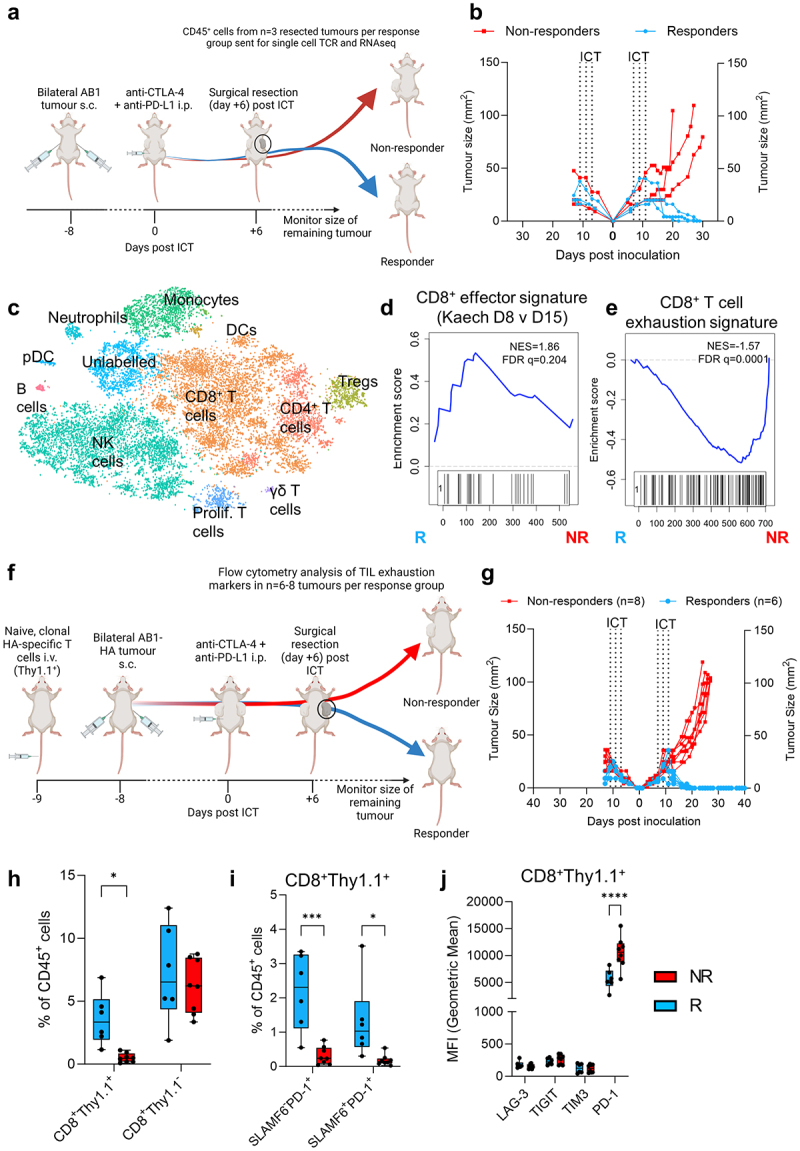
Clonally expanded CD8^+^ TILs have displayed an effector and progenitor exhausted phenotype. (a) Experimental schematic for single cell experiment (b) AB1 tumor growth curves of ICT (anti-CTLA-4 + anti-PD-L1) treated animals (*n* = 3/group). Dotted lines on growth curves represent when ICT was administered. CD45^+^ T cells from right flank tumor excised 6 days post ICT sent for single cell analysis. (c) TSNE clustering of all 18,029 cells, labeled by color and cell type. (d) GSEA plot depicting significant enrichment of an effector T cell signature in CD8^+^ large clones from responding tumors, (e) Enrichment of T cell exhaustion signature in CD8^+^ T cells from non-responding tumors. Negative NES indicates enrichment in non-responders. (f) Experimental schematic for flow cytometry experiment in AB1-HA bearing animals with HA-specific, CL4 TCRβ transgenic (CD8^+^Thy1.1^+^) T cells. (g) AB1-HA tumor growth curves of ICT (anti-CTLA-4 + anti-PD-L1) treated animals (*n* = 6–8/group). (h) Frequencies of HA-specific (CD8^+^Thy1.1^+^) and endogenous (CD8^+^Thy1.1^−^) TILs, (i) frequencies of effector (SLAMF6^−^PD-1^+^), progenitor exhausted (SLAMF6^−^PD-1^+^) HA-specific (CD8^+^Thy1.1^+^) TILs, and (j) MFI of inhibitory receptor expression on HA-specific (CD8^+^Thy1.1^+^) TILs in responding and non-responding tumors. Mann-Whitney tests used to compare between responder and non-responder groups at individual timepoints. *p < 0.05, ***p < 0.0005.

We next characterized the transcriptome of clonally expanded T cells from AB1 tumors 6 days post ICT using 5’ single cell RNA and TCR sequencing on sorted CD45^+^ cells. We performed another surgical experiment in which tumors from one flank were excised while the other was used as a readout for response (Figure 5(a,b)). Cell clusters were assigned to cell types including CD8^+^, CD4^+^ T cells, Foxp3^+^ regulatory T cells, monocytes, NK cells and macrophages (Figure 5(c)) with variable cell numbers and proportions between samples (Supp Figure S6A, B). There was no significant difference between responders and non-responders in overall annotated cell proportions in this analysis (Supp Figure S6(a)). We focused on clonally expanded TILs because oligoclonal expansion is a feature of post ICT TILs in our models.^23^ Large clones, defined as cells with identical TCRα/β sequences ≥ 1.0% of the total number of TCR expressing cells, were almost exclusively CD8^+^ T cells (Supp Figure S6C). We compared differentially expressed genes (DEGs) between large clones from non-responding versus their counterparts from responding tumors. Pathway analysis of DEGs shows that clonally expanded TILs were enriched in genes from proinflammatory, TNF signaling pathways in non-responders (Supp Figure S6D). Clonally expanded TILs from responding tumors were enriched in genes from cell cycle and proliferation hallmark pathways (Supp Figure S6E). Using gene set enrichment analysis, we compared our gene expression data against published mouse CD8^+^ T cell and TIL gene sets, and found that non-responders were significantly enriched for a T cell exhaustion gene signature for both large and other clones (Figure 5(d)). Large clones from responding tumors were enriched for an effector CD8^+^ T cell signature compared to non-responders, but this enrichment was not observed when other clones were compared (Figure 5(e)).

As single cell analysis was limited by sample size and variation in cell numbers between samples, we used flow cytometry to assess CD8^+^ T cell effector and exhaustion markers. We seeded animals with HA-specific CL4 TCR transgenic T cells. CL4 TCR transgenic CD8^+^ T cells expressed allelic marker Thy1.1, which allowed us to track clonally expanded, tumor antigen (HA)-specific CD8^+^ T cells by flow cytometry in AB1-HA (Figure 5(f)). We treated animals with combination ICT, and surgically excised tumors from one flank post ICT at the same schedule as the single cell experiment. Responders and non-responders to ICT were similarly observed (Figure 5(g)). Responding tumors were enriched with clonal population of HA-specific T cells (CD8^+^Thy1.1^+^), but not the endogenous CD8^+^Thy1.1^−^ populations, suggesting clonally expanded HA-specific T cells are present in responding AB1-HA tumors (Figure 5(h), Supp Figure S7A).

Responding AB1-HA tumors had increased frequencies of progenitor exhausted (PD-1^+^TIM3^−^SLAMF6^+^) and effector (PD-1^+^TIM3^−^SLAMF6^−^) CD8^+^Thy1.1^+^ T cells (Figure 5(i), Supp Figure S7B), suggesting that clonally expanded HA-specific T cells were enriched with an effector and progenitor exhausted signature in responders. For T cell exhaustion associated genes that were enriched in non-responders from our single cell data, CD8^+^Thy1.1^+^ HA-specific T cells in non-responders had higher expression levels of PD-1 compared to responders, but not TIM3, TIGIT and LAG3 (Figure 5(j), Supp Figure S7C). Differences in effector, progenitor exhausted T cell frequencies and inhibitory receptor expression between response groups were not observed in the endogenous CD8^+^Thy1.1^−^ T cell population (Supp Figure S7D-G). Clonally expanded TILs from responders were enriched with an effector CD8^+^ T cell gene signature and phenotype.

### ICT responders exhibit early clonal expansion of clonotypes associated with tumour antigen-specificity

**Figure 6.**
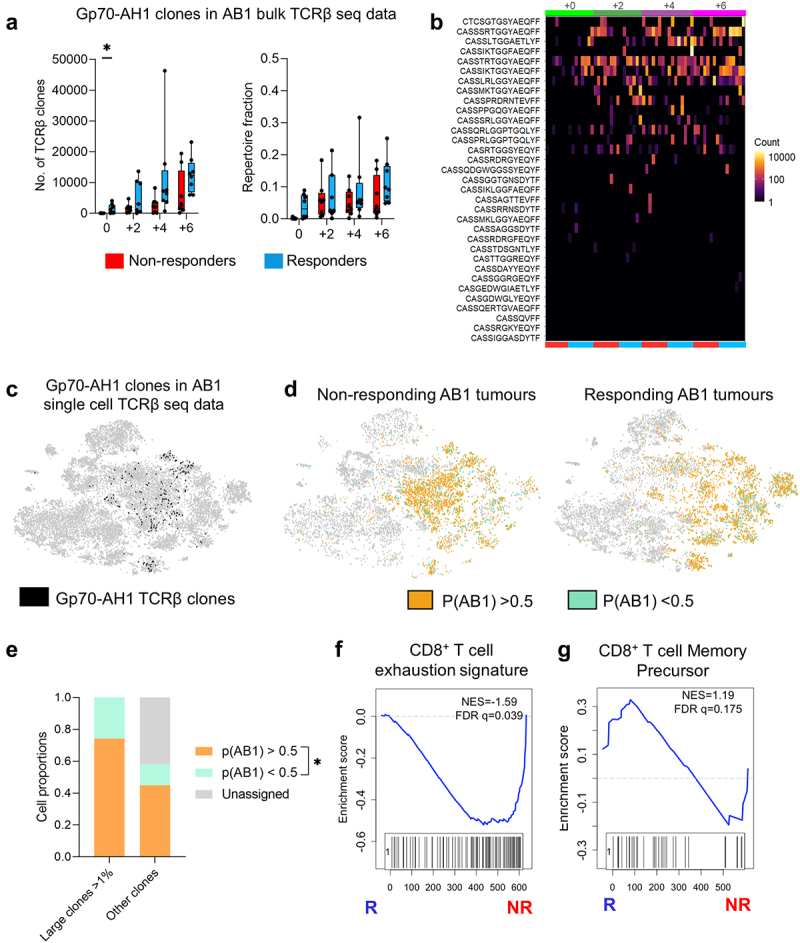
Tumor antigen-specific TCRβ clonotypes increase earlier in time in ICT responders. (a) The number, and proportion of gp70-AH1 associated TCRβ clones in ICT responding and non-responding AB1 tumors across time. (b) The distribution of each gp70-AH1 associated TCRβ clone across individual animals represented as a heatmap. Each rectangle on the heatmap represents an individual animal at a particular time points, and ICT response. (c) The number of cells with gp70-AH1 antigen specific TCRβ clonotypes in the single cell dataset, with (d) p(AB1) high (>0.5) and low (<0.5) cells depicted on a tSNE plot from single cell analysis of day 6 ICT. (e) Proportion of large or other clones that have TCRβs with either p(AB1) high and low scores. (f,g) GSEA plots depicting enrichment of T cell exhaustion or memory precursor gene signatures in pAB1 high cells, responders vs non-responders. Positive NES indicates enrichment in responders. Kruskal Wallis tests were used to compare between timepoints. Multiple mann-whitney tests were used to compare between responder and non-responder groups. All tests were adjusted for multiple comparisons with a false discovery rate of 0.1. *q < 0.05. Friedman tests were used to compare cell populations from matched samples. *p < 0.05.

Since expansion of model tumor (HA) antigen-specific TCRβ clones associated with ICT response, we searched public TCR databases for endogenous tumor antigen-specific TCRβ clonotypes (Supp Table 4). The murine leukemia virus envelope glycoprotein gp70 is a tumor-associated self-antigen expressed in BALB/c-derived tumor cell lines including AB1.^41^ We tracked 60 unique and public TCRβ CDR3 sequences derived from sorted H2-Ld restricted gp70_423–431_ (gp70-AH1) specific CD8^+^ TILs.^42,43^ In our bulk TCRβ sequencing data set, TCRβ clones with exact TRBV, TRBJ and CDR3 region matches with gp70-AH1 clones significantly increased over time in AB1 tumors and were dominant in some tumors, making up approximately 10–30% of the TCRβ repertoire (Figure 6(a)). Responding AB1 tumors had more gp70-AH1 TCRβ clonotypes before treatment than non-responding tumors, suggesting that expansion of tumor associated antigen gp70-AH1 specific T cell clones contributed to the difference in TCRβ repertoire diversity between responders and non-responders in the AB1 model (Figure 6(a)). Some individual gp70-AH1 TCRβ clones were distributed randomly between mice, suggesting that some of these clones were public (Figure 6(b)). We also found 224 cells expressing gp70-AH1 associated TCRβ genes in our TIL single cell data set from the AB1 model, and > 90% of these gp70-AH1 cells were large clones (Figure 6(c)).

We also assessed the overlap between our single cell and bulk TCRβ data. Large TCRβ clones from the single cell data were present to varying numbers and proportions in the bulk AB1 TCRβ data (ranging from 0–12.2% of each mouse’s repertoire). The overlap was significantly higher with AB1 than RENCA TCRβ bulk data, suggesting that shared TCRβ clones unique to AB1 tumor model (Supp Figure S8A, SB). We applied the DeepTCR AB1 model related signature trained on bulk TCRβ data onto single cell data. 74.2 ± 0.07% of large clones in the single were high in p(AB1) probability score (>0.5), compared to 2.58 ± 0.07% of large clones that were low in p(AB1) probability score (<0.5) (Figure 6(d,e)), suggesting that most of the clonally expanded TILs were TCRβs unique to the AB1 tumor model. Cells with high p(AB1) scores in non-responding tumors were enriched with a T cell exhaustion gene signature^33^ (Figure 6(f)). p(AB1) high cells in responding tumors were enriched with a previously described CD8^+^ T cell memory precursor gene signature^32^ (Figure 6(g)).

Taken together, responding animals displayed a more rapid oligoclonal expansion of TCRβ clonotypes that are possibly tumor-specific, while decreasing in overall TCRβ diversity compared to non-responding animals. Responding animals displayed clonal expansion of TILs with an effector phenotype. By applying neural networks to TCR sequencing data, we found overlapping TCRβ signatures that could delineate tumor models and ICT responses at selected timepoints.

## Discussion

ICT increases T cell infiltration into tumors, and the clonal composition of TIL repertoires can be measured by TCRβ sequencing. Oligoclonal expansion of TILs post ICT is often indicative of antigen-specific T cell activation and proliferation, which is reflected by a decrease in tumor TCRβ diversity.^42,44,45^ Differences in TCRβ diversity of responding and non-responding tumors were previously found in some clinical studies,^11,46,47^ but not in others.^13,48,49^ However, most studies were limited to a single measurement of TCRβ diversity because frequent serial tumor biopsies are not feasible in solid cancers. Our group and others have highlighted the utility of bilateral tumor models to track anti-tumor responses because of the high fidelity of T cell repertoires within the same animal.^50,51^ For these reasons, we studied mouse models that allow tumor sampling at multiple time points during the response to ICT.

ICT responding murine tumors were characterized by a decrease in TCRβ diversity and an increase in clonality earlier in time than non-responding tumors. This supports findings that reduction of TIL TCRβ diversity in paired biopsies before and after ICT correlated with increased overall survival or responses to ICT.^11–13,46,48^ Although changes in peripheral blood TCRβ diversity were not detectable in our models, an increase in peripheral blood TCRβ clonality from pre- to 3 weeks post-ICT was associated with a positive outcome in some clinical trials.^35,37^ Peripheral T cell repertoires are often characterized by many unique TCRβ clones as compared to tumor or organs. We speculate clonal expansion was not large enough to noticeably alter overall repertoire metrics in this study because overall diversity and clonality metrics are resistant to changes caused by individual or a few clones. Detailed analyses of peripheral blood TCRβs, such as tracking shared clones derived from tumor sequencing data, or tracking TCRβs of T cells expressing activated phenotypes in blood will be more informative of ICT response than bulk TCRβ analysis of whole blood samples.

In line with previous work that derived ICT predictive TCRβ signatures from machine learning of clinical data,^26^ we found TCRβ signatures associated with ICT response in murine tumors. ICT response signatures could only be trained from pre-ICT data in one model (AB1), and post-ICT (day 4) in the other (RENCA). Differences between tumor models could be attributed to antigen profiles. For example, a strong antigen in AB1 might result in stable expression of tumor-related TCRβs post-ICT regardless of response. Another possibility is that intra-tumoral interferon signaling is temporally different between responders and non-responders in both models, resulting in different TCRβ clonotype expansion patterns over time.^39^ Our study highlights the challenge of timepoint selection for developing a TCRβ based biomarker, as DeepTCR was unable to derive a response associated signature at majority of the timepoints studied. The number and type of tumor antigens present in mouse models will differ from human tumor antigens, and a caveat of our study is that the models express strong antigens.^52^ Although we observed oligoclonal expansion with ICT that is similar to clinical studies, further work is required to understand how differences in tumor antigens are reflected in the TIL TCRβ repertoires. In-depth understanding of T cell dynamics, particularly at early timepoints after initial cycles of therapy is required to inform the optimal timing to sample for biomarker development. There is also a need to develop accessible, deep learning algorithms to integrate transcriptomics, tumor genomic data and TCRβ data to help derive response signatures.

A decrease in TCRβ diversity associated with ICT response was attributed to clonal expansion of tumor-antigen specific T cells. Oligoclonal expansion of gp70-AH1 specific T cells in AB1 and CT26 murine cancer models were similarly observed after different therapies including anti-CTLA-4 and anti-PD-L1.^41,42^ Oligoclonal expansion of TILs independently of clinical response to ICT might predict that the differentiation status of TILs, such as T cell effector or exhaustion would be different in responders versus non-responders.^7,53^ Indeed, clonally expanded CD8^+^ TILs from responding tumors were enriched with an effector and progenitor exhausted phenotype.^33^ Further studies are required to elucidate whether TIL differentiation can be incorporated into machine learning classification of TCRβs in relation to ICT outcomes.

Mapping TCRβ repertoire dynamics during other therapies is of great interest, especially if they are administered in combination with ICT. Immunogenic chemotherapies that improve tumor-antigen cross-presentation could increase tumor clonal expansion and reduce tumor TCRβ diversity, favoring ICT responses.^54^ In addition to the expression of T cell antigenic targets, T cell phenotype, and strength of T cell responses, we provide a strong rationale for mapping the dynamics in T cell repertoires as an important way forward for understanding and improving the response to ICT.

## Supplementary Material

Supplemental Material
