# Supplementary figures and images for: Immune checkpoint therapy responders display early clonal expansion of tumor infiltrating lymphocytes
Source: Oncoimmunology. 2024 Apr 26;13(1):2345859. doi: 10.1080/2162402X.2024.2345859 (PMC11057660; doi:10.1080/2162402X.2024.2345859)

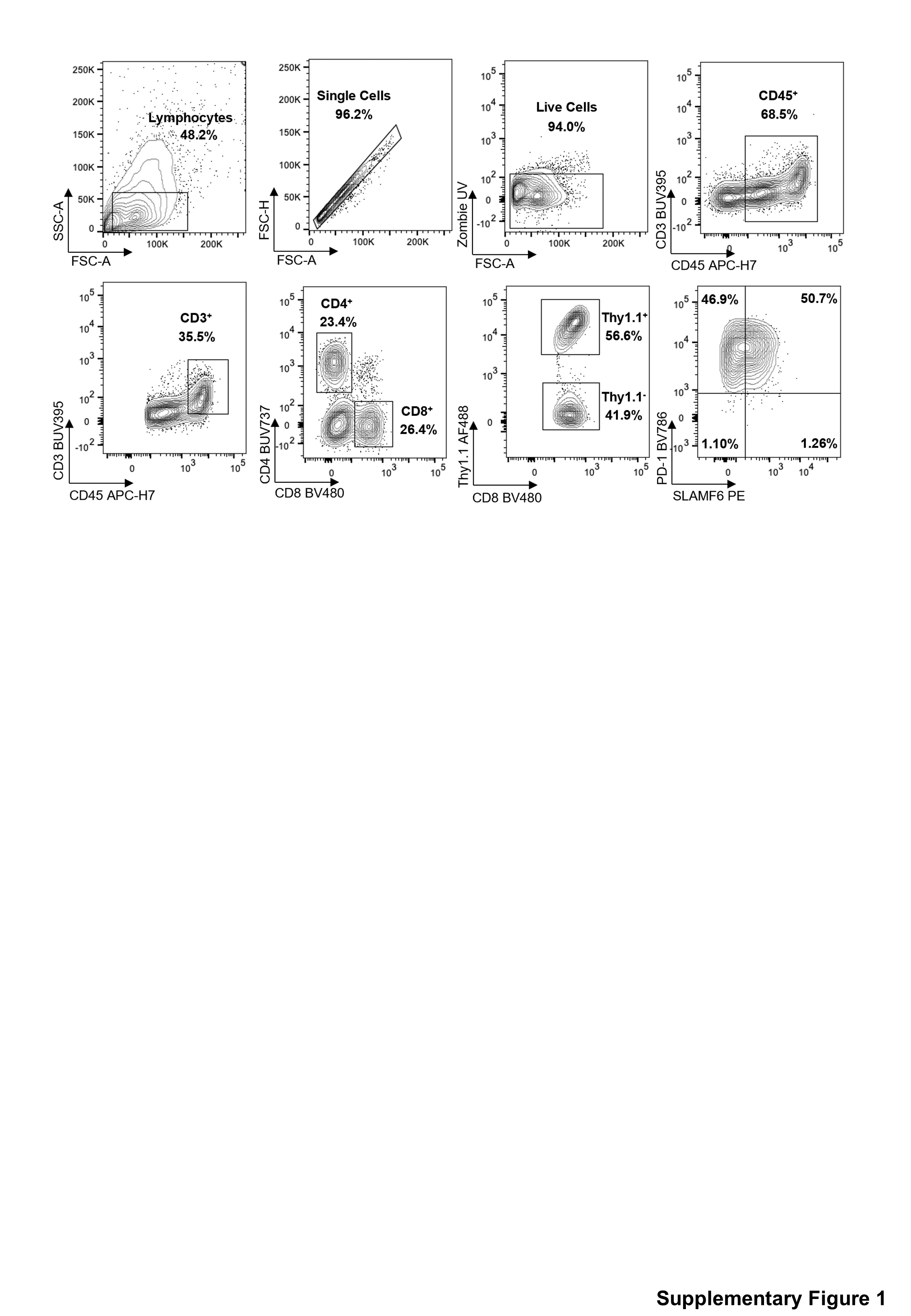

Supplement: Supplemental Material [file KONI_A_2345859_SM4454.zip › New folder (2)/FigS1.PNG]

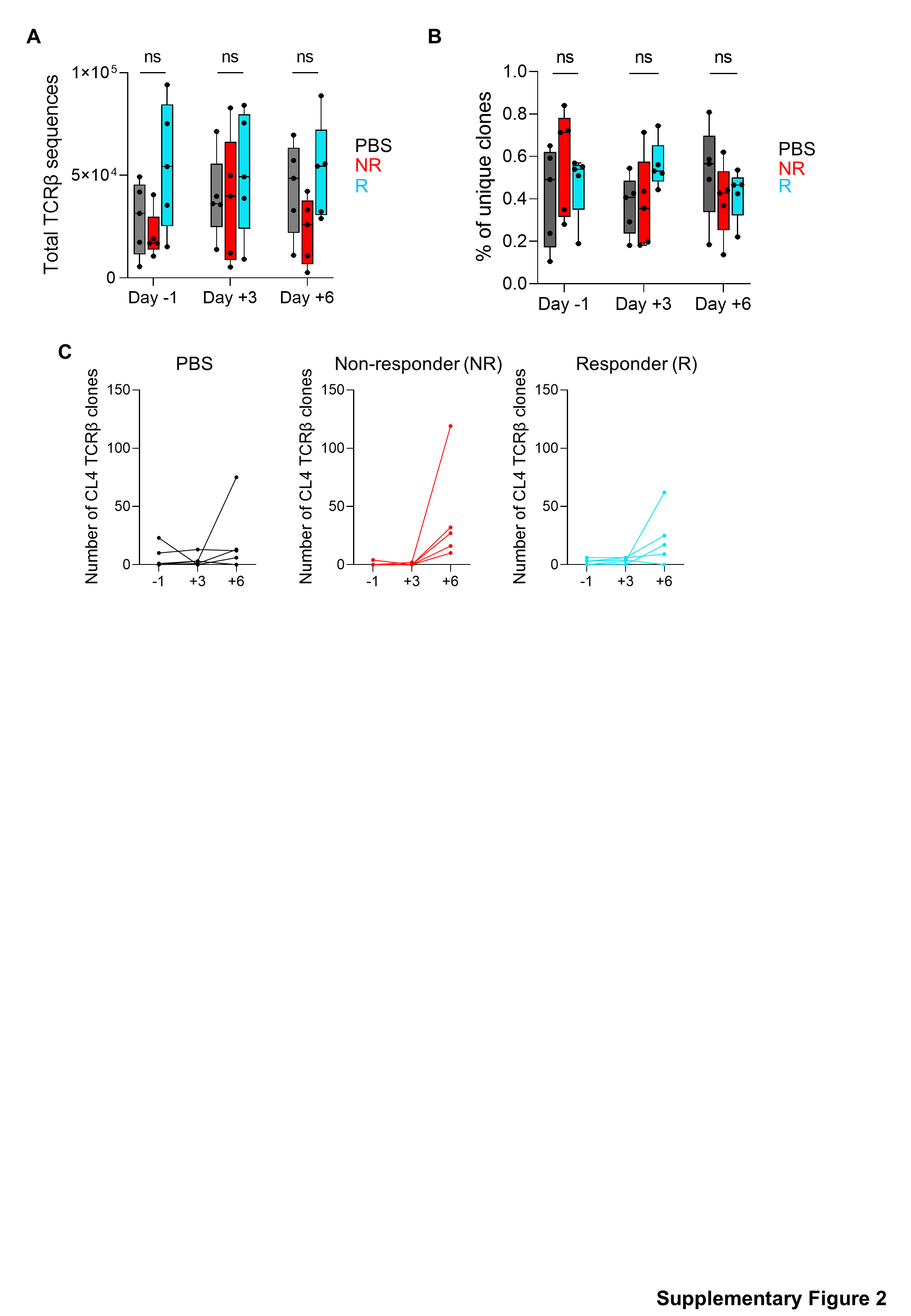

Supplement: Supplemental Material [file KONI_A_2345859_SM4454.zip › New folder (2)/FigS2.PNG]

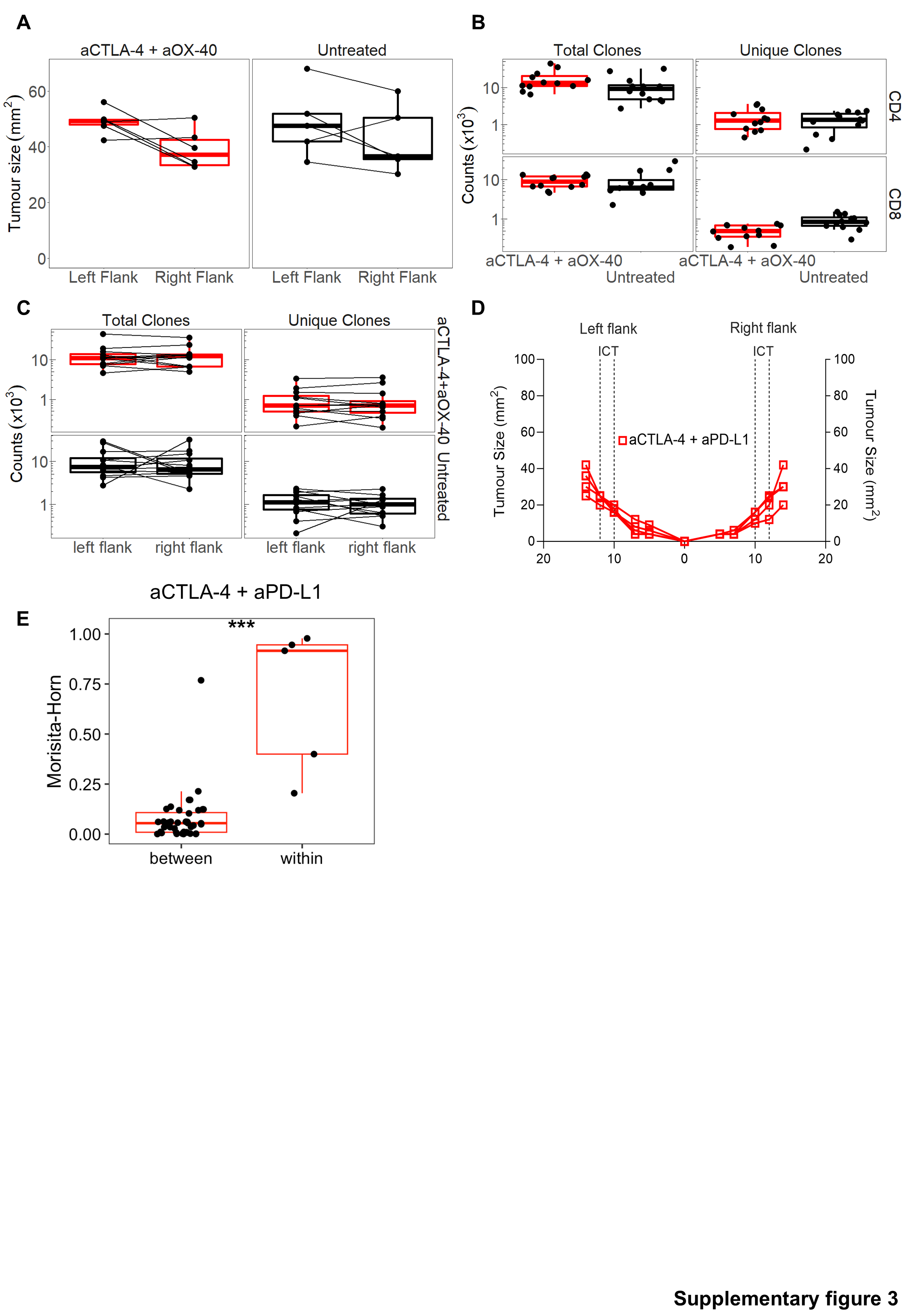

Supplement: Supplemental Material [file KONI_A_2345859_SM4454.zip › New folder (2)/FigS3.PNG]

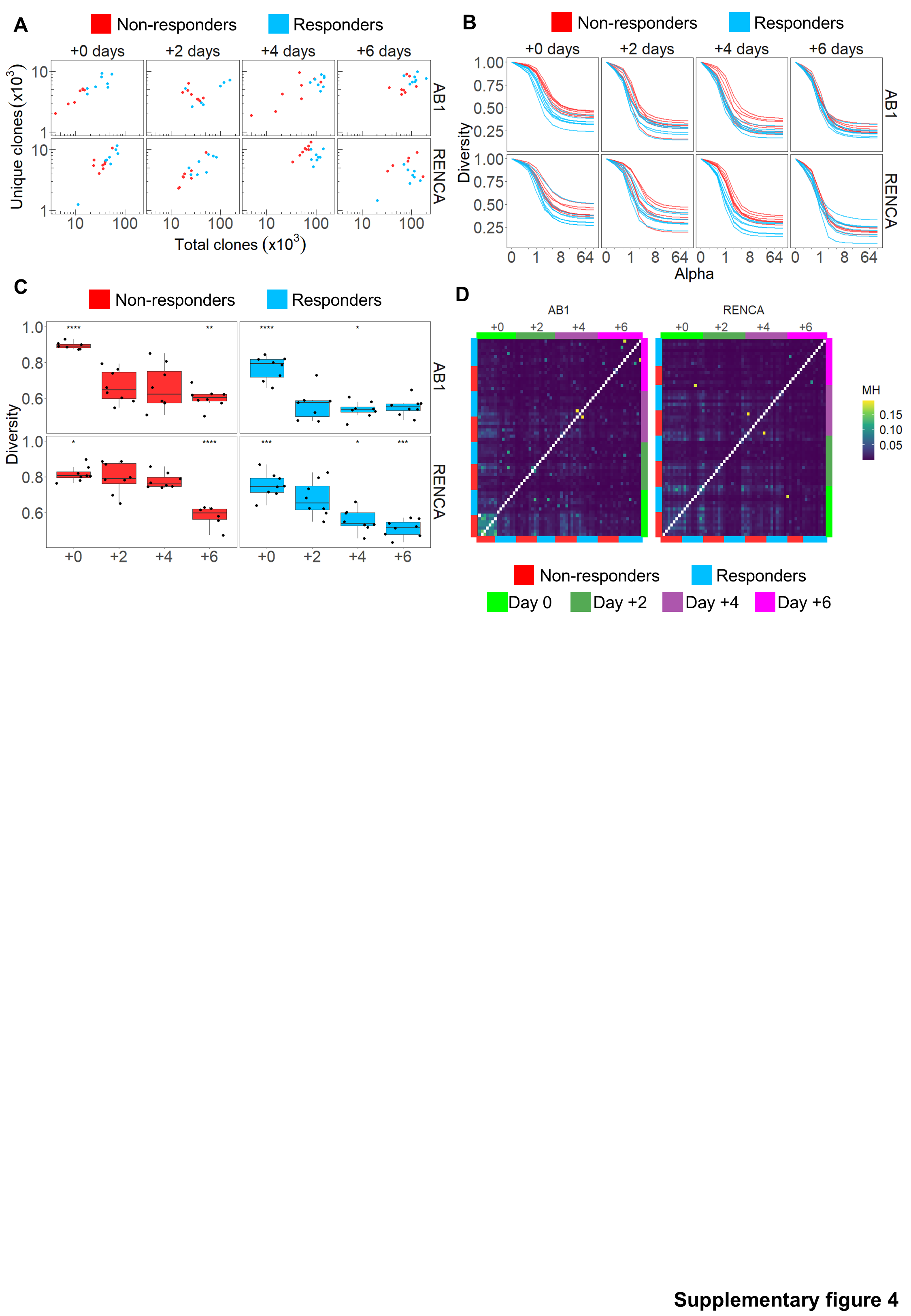

Supplement: Supplemental Material [file KONI_A_2345859_SM4454.zip › New folder (2)/FigS4.PNG]

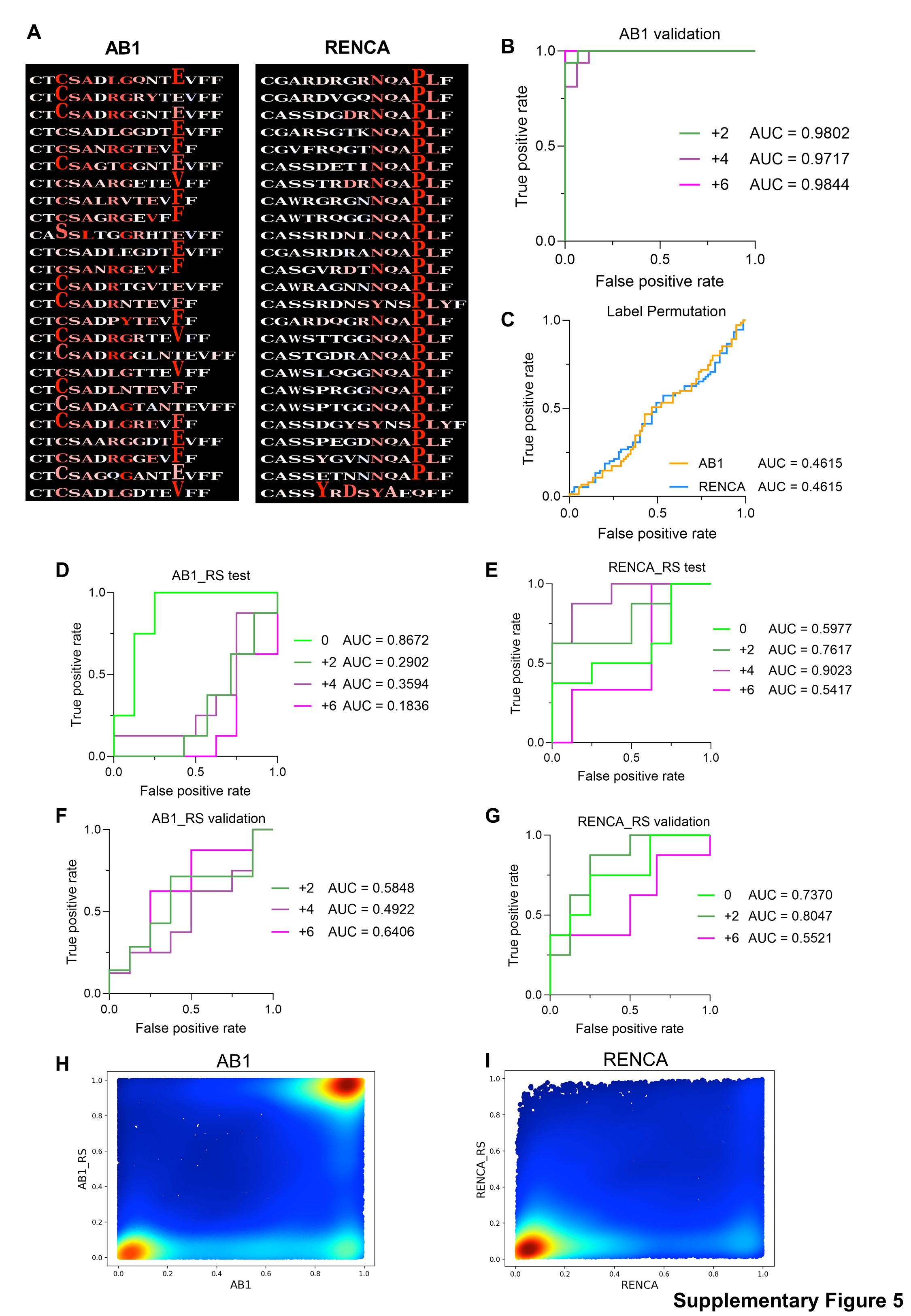

Supplement: Supplemental Material [file KONI_A_2345859_SM4454.zip › New folder (2)/FigS5.PNG]

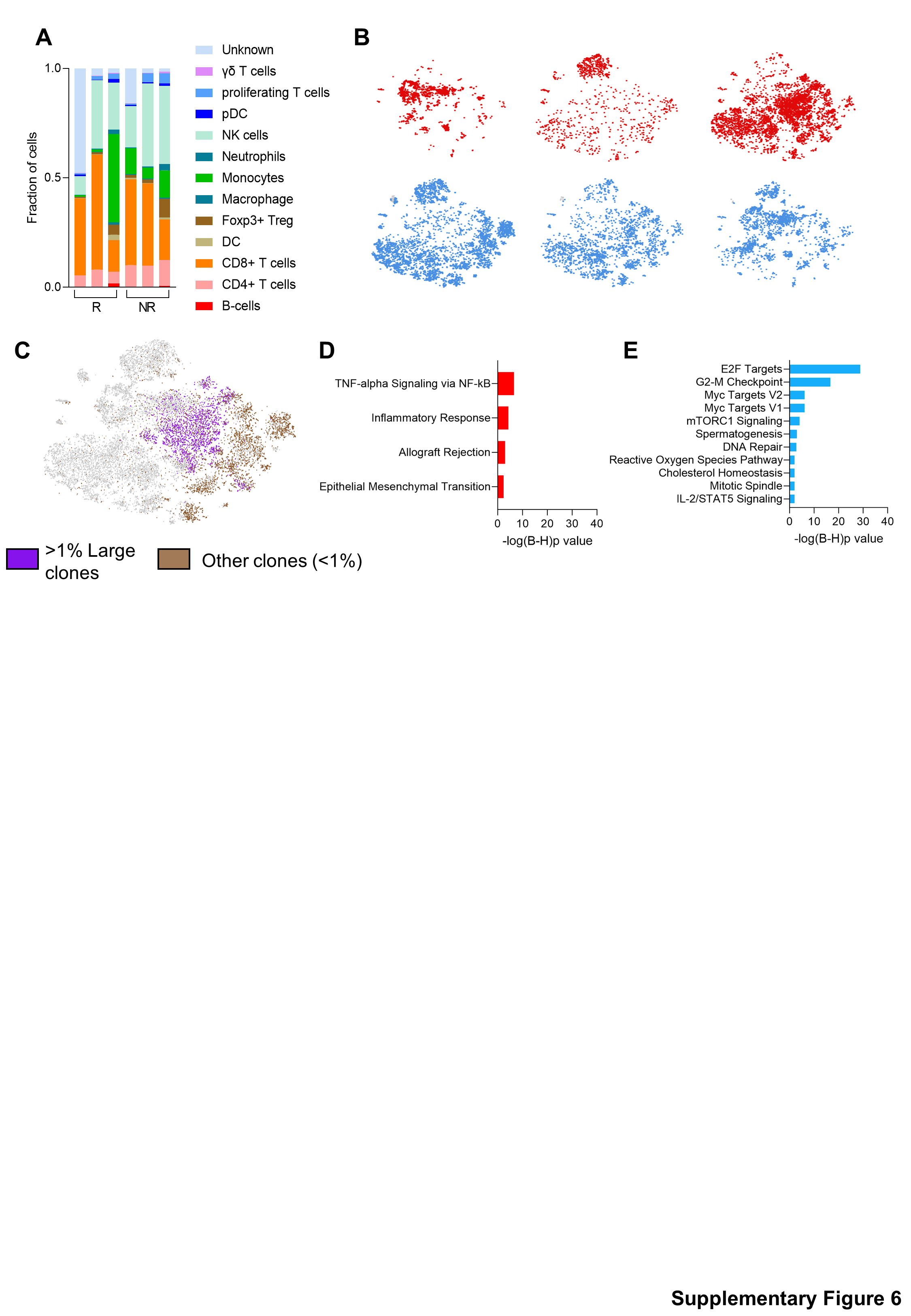

Supplement: Supplemental Material [file KONI_A_2345859_SM4454.zip › New folder (2)/FigS6.PNG]

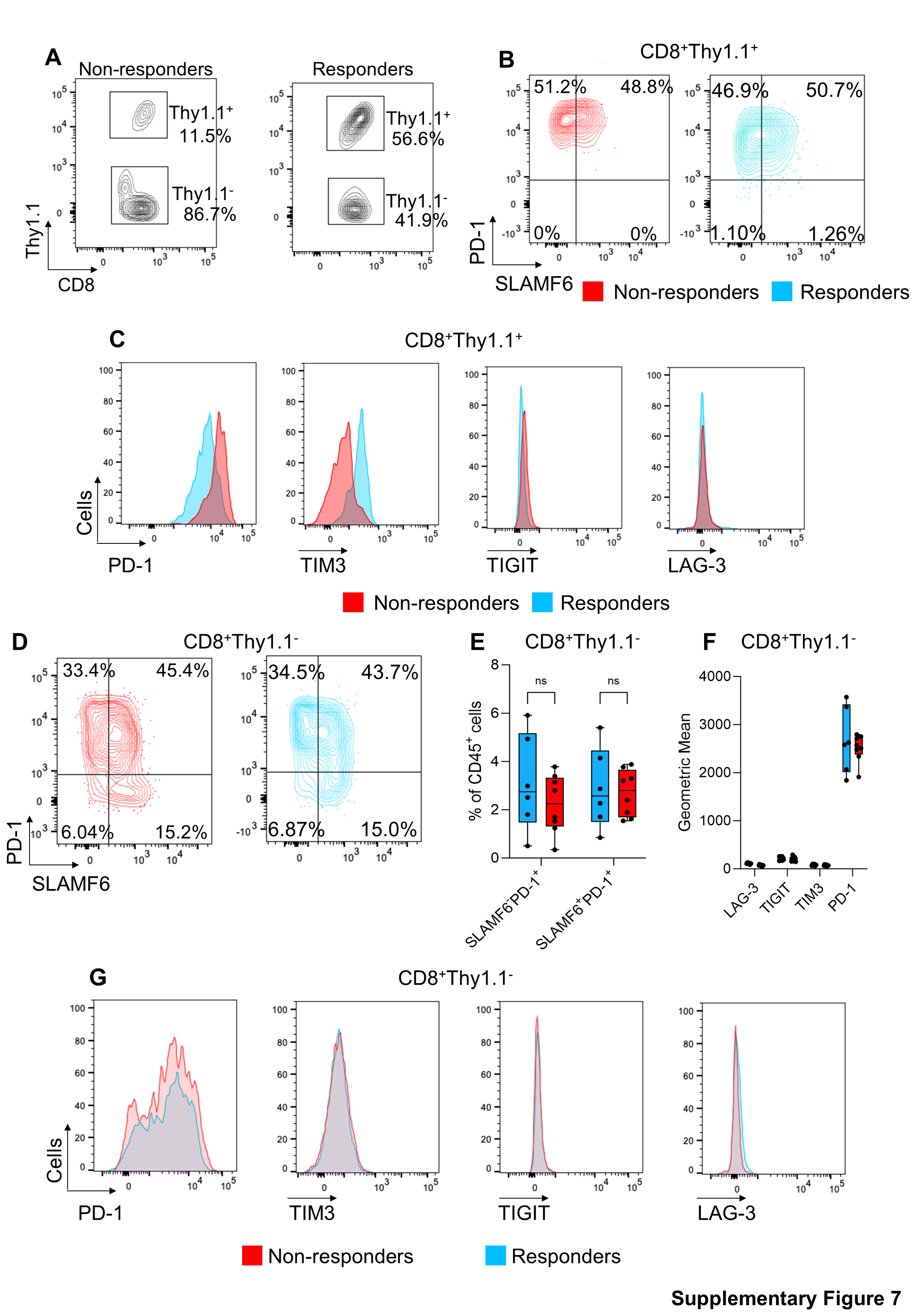

Supplement: Supplemental Material [file KONI_A_2345859_SM4454.zip › New folder (2)/FigS7.PNG]

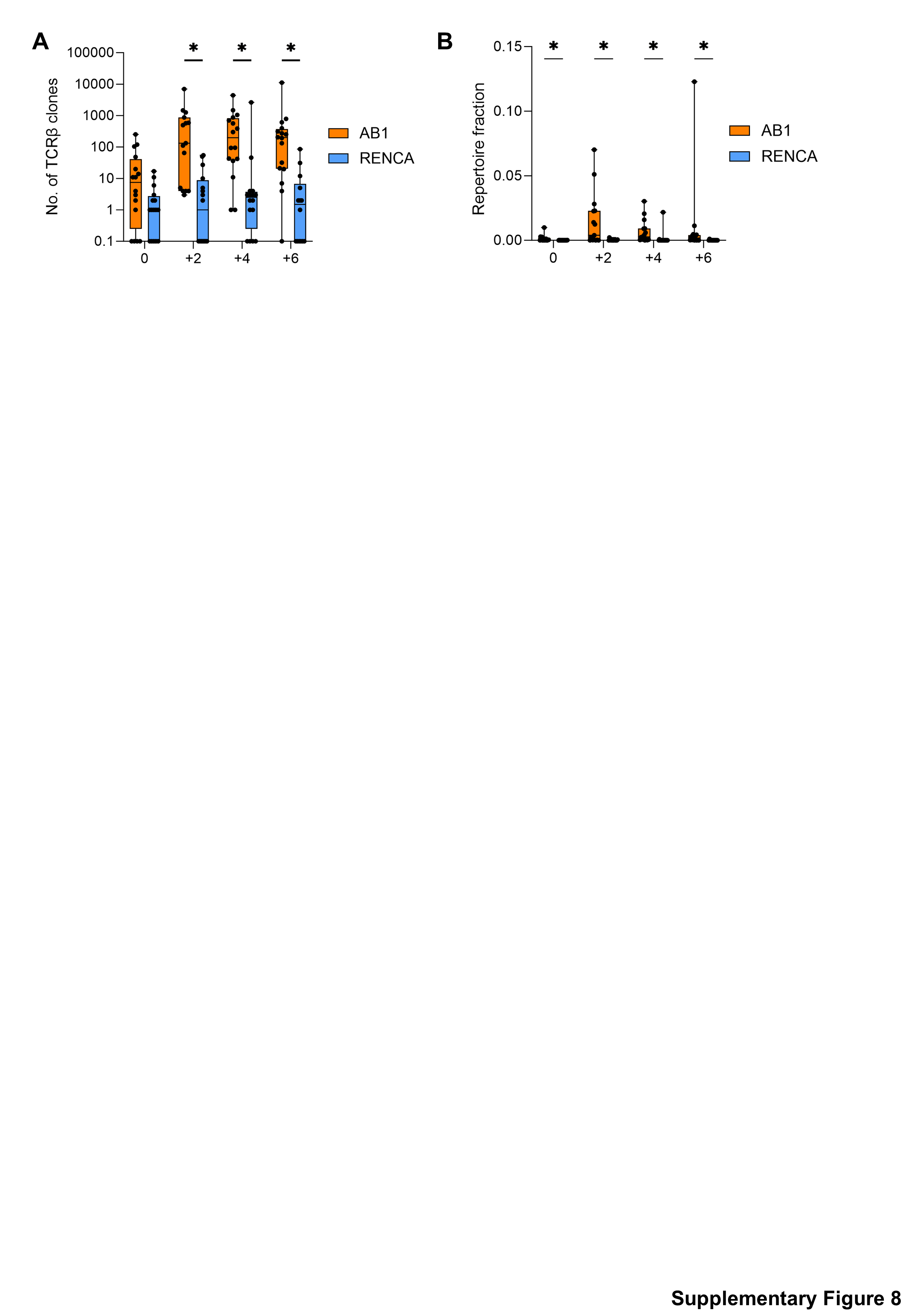

Supplement: Supplemental Material [file KONI_A_2345859_SM4454.zip › New folder (2)/FigS8.PNG]
